# Experiments with Various Plastic Filling Materials

**Published:** 1883-08

**Authors:** 


### EXPERIMENTS WITH VARIOUS PLASTIC FILLING MATERIALS.

In order to determine the resistance to acids of various filling materials, the following experiments were made:

1st. Two grammes of Fletcher's white enamel were put into a flask containing a small quantity of sour milk, on September 6th. The reaction was acid and continued so until December 6th, when the filling material had lost 1.2 grammes in weight.

2nd. On September 6th, two grammes of Hill's Stopping were put into thirty grammes of common vinegar. The reaction continued to be acid for three months. But at the end of this time the filling material had disappeared, and the only evidence of its presence consisted in a thin film floating on the liquid. The stopping had been entirely dissolved in the dilute acetic acid. This would indicate that people who wear a rubber plate should always use an antacid tooth powder or mouth-wash after a meal containing vinegar in any shape.

3d. Two grammes of Superior Cement, made by Ash, were put into a small quantity of sour milk, on September 6th. Acid reaction all through to December 6th. Loss in weight 0.2 gramme.

4th. Poulson's new mineral cement put into sour milk for the same length of time as above, suffered a loss in weight of 0.3 grammes.—H. Spoerl in *Monatsschrift des Vereins Deutscher Zahnkuenstler.*

